# MicroRNAs in Acute COVID‐19 and Long COVID: Dysregulation, Pathogenic Roles, and Clinical Implications

**DOI:** 10.1155/jimr/5862241

**Published:** 2026-04-24

**Authors:** Larissa I. Silva, Carlos M. Gonzalez-Zambrano, Vanessa C. M. P. Ferreira, Fernando C. Corrêa, Luciane A. Dias-Melicio

**Affiliations:** ^1^ Laboratory of Immunopathology and Infectious Agents - LIAI - UNIPEX - Experimental Research Unity (UNIPEX - Sector 5), Medical School of Botucatu (FMB), São Paulo State University (UNESP), Botucatu, São Paulo, Brazil, unesp.br; ^2^ Department of Structural and Functional Biology, Botucatu Biosciences Institute (IB), São Paulo State University (UNESP), Botucatu, São Paulo, Brazil, unesp.br; ^3^ Department of Pathology, Medical School of Botucatu (FMB), São Paulo State University (UNESP), Botucatu, São Paulo, Brazil, unesp.br

**Keywords:** biomarkers, COVID-19, inflammation, long COVID, microRNAs

## Abstract

MicroRNAs (miRNAs) are key post‐transcriptional regulators of gene expression with central roles in immune responses, inflammation, and viral pathogenesis. Increasing evidence indicates that severe acute respiratory syndrome coronavirus (SARS‐CoV‐2) infection induces marked dysregulation of host and viral miRNAs (v‐miRNAs), contributing to disease severity during acute COVID‐19 and to the persistent manifestations observed in long COVID (LC). This narrative review critically synthesizes current evidence on miRNA dysregulation across the acute and post‐acute phases of COVID‐19, highlighting their pathogenic roles, clinical relevance, and existing knowledge gaps. During acute infection, altered miRNA profiles—including those associated with immune activation, endothelial dysfunction, and immunothrombosis—reflect both host responses and viral strategies of immune modulation, including miRNAs carried by extracellular vesicles (EVs) and SARS‐CoV‐2–derived v‐miRNAs. In LC, emerging data suggest that persistent miRNA alterations are associated with unresolved inflammation, pulmonary dysfunction, neurological symptoms, and vascular injury, although available studies remain limited and heterogeneous. Overall, miRNAs represent promising biomarkers and potential therapeutic targets in COVID‐19; however, robust longitudinal and mechanistic studies are urgently needed to clarify their causal roles and translational utility in post‐acute disease.

## 1. Introduction

### 1.1. Epidemiology and Clinical Definitions

In December 2019, clusters of patients in Wuhan, China, were hospitalized with pneumonia of unknown etiology, later identified as being caused by a novel coronavirus closely related to severe acute respiratory syndrome coronavirus (SARS‐CoV‐2). Early epidemiological and virological investigations rapidly confirmed efficient human‐to‐human transmission and a distinct mechanism of host cell entry mediated by the angiotensin‐converting enzyme 2 (ACE2) receptor [1]. Since then, SARS‐CoV‐2 has evolved into a global public health crisis, resulting in hundreds of millions of confirmed cases and millions of deaths worldwide, placing unprecedented strain on healthcare systems and societies globally [2].

As a positive‐sense RNA virus, SARS‐CoV‐2 has undergone continuous genetic evolution driven by its intrinsic mutation rate and selective immune pressure. Variants of concern (VOCs), including Alpha, Gamma, Delta, and Omicron, harbor critical mutations in the spike glycoprotein that influence viral transmissibility, immune escape, and, to varying degrees, vaccine effectiveness [3]. Genomic and phylogenetic analyses indicate that SARS‐CoV‐2 adaptation reflects progressive optimization for human transmission under selective pressure from neutralizing antibodies and population‐level immunity [4]. Importantly, beyond the acute phase of infection, large multinational cohorts of hospitalized patients have demonstrated that COVID‐19 continues to impose a substantial burden of morbidity and mortality, with a significant proportion of survivors developing persistent post‐acute sequelae, now collectively referred to as long COVID (LC) [4, 5].

**Figure 1:**
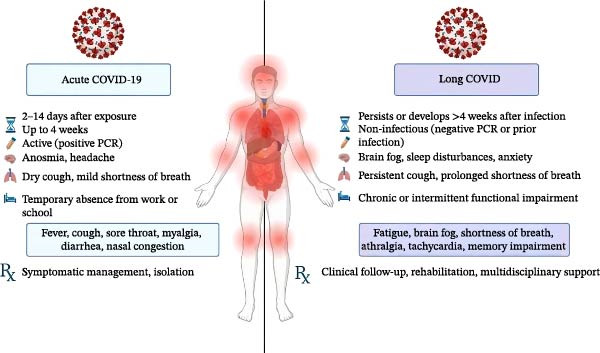
Differential clinical manifestations in acute COVID‐19 and long COVID. This figure illusclinical differences observetrates the key clinical differences between patients during the acute phase of COVID‐19 and those experiencing persistent or late‐onset symptoms (long COVID). The comparison includes symptom duration, predominant clinical manifestations, severity, and potential complications, highlighting the progression from an acute viral infection to a prolonged multisystem condition in some individuals.

Acute COVID‐19 typically manifests with respiratory symptoms ranging from mild upper airway involvement to severe pneumonia and acute respiratory distress syndrome (ARDS) [1, 6]. In contrast, LC, also referred to as post‐acute sequelae of COVID‐19 (PASC), is defined by the persistence or emergence of symptoms beyond 4–12 weeks after infection, including fatigue, dyspnea, cognitive impairment, dysautonomia, and thromboinflammatory complications [2, 5]. The differential clinical manifestations in COVID‐19 and LC are shown in Figure 1.

### 1.2. Pathophysiology of Acute COVID‐19

SARS‐CoV‐2 infection triggers a complex and tightly regulated immune response involving both innate and adaptive immunity [6]. Activation of pattern recognition receptors, such as TLR3, TLR7, and RIG‐I, leads to interferon production and inflammatory signaling, while adaptive responses include CD4^+^ and CD8^+^ T‐cell activation and neutralizing antibody generation [7]. Dysregulation of these processes—characterized by lymphocyte exhaustion, hypercytokinemia, endothelial dysfunction, excessive cytokine production (IL‐6 and TNF‐α), and coagulation abnormalities—is strongly associated with severe disease and multisystem involvement [8–10]. Host genetic factors, including polymorphisms in ACE2, TMPRSS2, and the 3p21.31 locus, further modulate susceptibility and clinical outcomes [10–12]. These mechanisms not only drive acute disease severity but also provide a biological framework for understanding the persistence of symptoms in LC. The imbalance between pro‐inflammatory and regulatory pathways is a central determinant of disease severity.

### 1.3. Pathophysiology of LC

Persistent symptoms may result from unresolved inflammation, endothelial dysfunction, microvascular injury, viral persistence, or immune dysregulation. Emerging data suggest that chronic low‐grade inflammation and impaired resolution pathways play central roles [2, 5]. However, the molecular mechanisms sustaining these alterations remain incompletely defined.

### 1.4. MicroRNAs (miRNAs) as Regulatory Integrators

Given the heterogeneity of clinical manifestations and the persistence of immune and inflammatory alterations after viral clearance, there is growing interest in molecular regulators capable of integrating viral, host, and environmental signals. Among these, miRNAs have emerged as central post‐transcriptional regulators of gene expression, with established roles in immune modulation, inflammation, metabolism, tissue repair, and viral infections [13, 14]. miRNAs are small non‐coding RNAs that regulate gene expression post‐transcriptionally by binding to complementary sequences in target mRNAs, leading to degradation or translational repression. Through fine‐tuning of signaling pathways, miRNAs regulate immune activation, RAAS components, endothelial integrity, and tissue remodeling. Their remarkable stability in plasma and other biological fluids, together with the availability of sensitive detection methods such as RT‐qPCR, has positioned circulating miRNAs as promising noninvasive biomarkers [15, 16].

In the context of COVID‐19, multiple studies have reported altered miRNA expression profiles during the acute phase of infection, with associations with disease severity, inflammatory burden, and clinical outcomes [15, 17]. More recently, emerging evidence suggests that persistent dysregulation of specific miRNAs may also contribute to the pathophysiology of LC, potentially reflecting sustained immune activation, metabolic imbalance, or impaired tissue recovery [18]. These miRNAs are often linked to pathways involved in cytokine signaling, endothelial function, and energy metabolism, highlighting their potential relevance not only as biomarkers but also as mechanistic mediators of post‐acute disease [14–18].

In this context, the present article is conceived as a narrative review aimed at synthesizing and critically discussing the available evidence on miRNA dysregulation in acute COVID‐19 and LC. The literature analyzed comprises original studies published between 2020 and mid‐2025 that evaluated miRNA expression in human samples during the acute and post‐acute phases of SARS‐CoV‐2 infection. Given the marked heterogeneity across studies—including differences in patient populations, disease severity, biological matrices, and analytical platforms, this review does not seek to perform a quantitative meta‐analysis, but rather to provide a biologically informed overview of recurrent miRNA signatures, their reported clinical associations, and their putative mechanistic relevance. Attention is given to methodological limitations and inconsistencies in the current literature, which constrain reproducibility and underscore the need for standardized and longitudinal investigations.

## 2. Methods

### 2.1. Study Design and Objective

This article is a narrative review designed to synthesize and critically discuss the current scientific evidence on dysregulated miRNAs in acute COVID‐19 and LC/post‐acute sequelae of SARS‐CoV‐2 infection (PASC). The primary objective was to explore the reported pathogenic and regulatory roles of miRNAs, their potential clinical relevance, and to identify methodological limitations and knowledge gaps that currently hinder causal interpretation and clinical translation.

### 2.2. Literature Search Strategy

A comprehensive literature search was conducted in the PubMed/MEDLINE database to identify relevant studies published between December 2019 and June 2025. The search strategy combined Medical Subject Headings (MeSH) terms and free‐text keywords using Boolean operators, as follows:

(“microRNA” OR “miRNA”) AND (“COVID‐19” OR “SARS‐CoV‐2”) AND (“Long COVID” OR “post‐acute COVID‐19” OR “PASC”).

(“microRNA” OR “miRNA”) AND (“COVID‐19” OR “SARS‐CoV‐2”) AND (“Long COVID” OR “post‐acute COVID‐19” OR “PASC”).

To assess the breadth of available literature, a broader search using the terms (“microRNA” AND “COVID‐19”) was also performed.

### 2.3. Search Results and Study Selection

The broad search strategy (“microRNA” AND “COVID‐19”) retrieved 762 records published from 2020 onward, reflecting the extensive body of literature addressing miRNA dysregulation during the acute phase of SARS‐CoV‐2 infection. In contrast, more specific searches incorporating post‐acute disease terminology retrieved substantially fewer studies, with 52 records identified using terms related to LC, post‐acute COVID‐19, or PASC, highlighting the relative scarcity of data on miRNA profiles in the post‐acute phase. After removal of duplicate records and screening for relevance based on titles and abstracts, studies were assessed for eligibility according to predefined inclusion criteria. Twenty‐seven original research articles, encompassing both acute COVID‐19 and LC, were ultimately included for qualitative synthesis.

### 2.4. Inclusion and Exclusion Criteria

#### 2.4.1. Studies Were Included According to the Following Criteria

Original research articles evaluating miRNA expression in the context of acute or post‐acute SARS‐CoV‐2 infection. Human studies measuring miRNA expression in biological samples, including plasma, serum, peripheral blood mononuclear cells (PBMCs), nasopharyngeal swabs, or tissue samples.

#### 2.4.2. Articles Published in English

Systematic reviews and meta‐analyses were included exclusively for background information, contextualization, and interpretation of findings, but were not considered as primary evidence in the qualitative synthesis. Studies exclusively based on in vitro or animal models, as well as editorials, commentaries, and opinion pieces, were excluded from the primary analysis but could be referenced for mechanistic context when relevant.

### 2.5. Data Extraction and Qualitative Synthesis

Eligible studies were analyzed qualitatively and categorized according to:

i. The phase of disease (acute COVID‐19 or LC).
ii. The specific miRNAs reported as dysregulated.
iii. Their proposed or experimentally supported molecular targets and signaling pathways.
iv. Any reported clinical associations, including disease severity, inflammatory markers, organ involvement, or persistent symptoms.

Given the substantial heterogeneity across studies—particularly in patient populations, disease severity, biological matrices, and analytical platforms, the synthesis was conducted in a descriptive and interpretive manner, rather than as a quantitative meta‐analysis. Emphasis was placed on identifying recurrent miRNA signatures, areas of biological convergence, and inconsistencies across reports, as well as highlighting methodological limitations that constrain reproducibility and causal inference.

**Figure 2:**
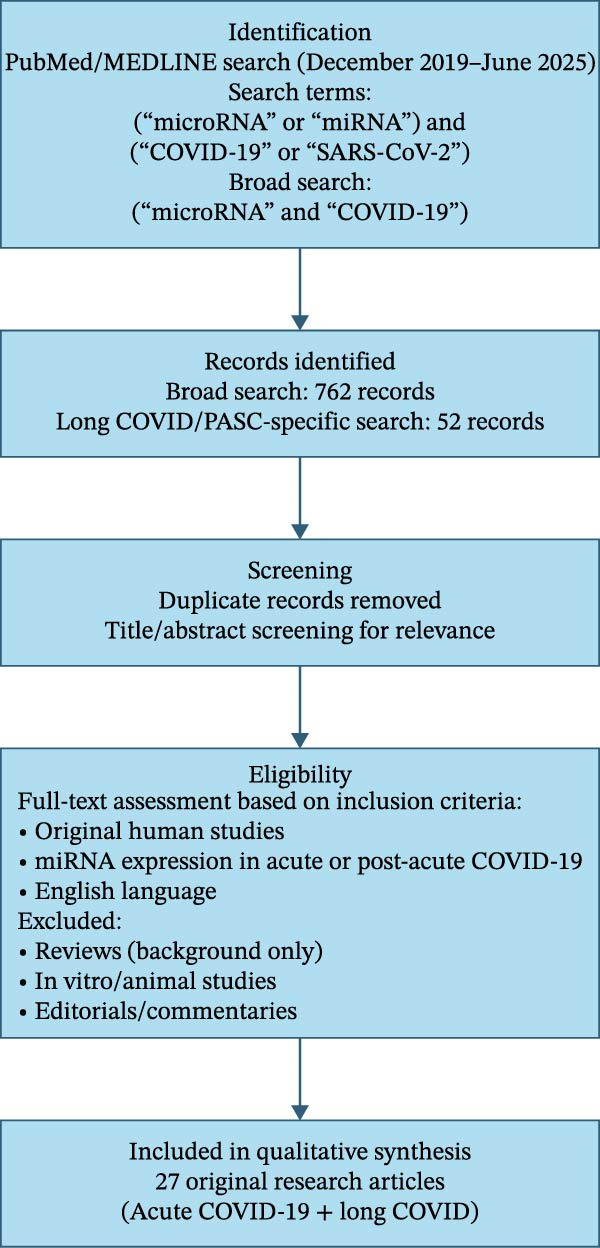
Study selection flowchart for the identification of studies on microRNAs in acute COVID‐19 and long COVID. This flowchart summarizes the identification, screening, eligibility assessment, and inclusion of studies evaluating microRNA expression in acute and post‐acute COVID‐19 (long COVID). The search was conducted in PubMed/MEDLINE December 2019 and June 2025 using predefined keywords. After removal of duplicate records and screening based on inclusion criteria, 27 original research articles were included in the qualitative synthesis.

The flow diagram of literature search and study selection is shown in Figure 2.

## 3. Results and Discussion

Since the onset of the COVID‐19 pandemic, numerous studies have demonstrated that SARS‐CoV‐2 infection induces widespread dysregulation of host miRNA expression across different tissues and biological fluids. These alterations consistently involve miRNAs regulating immune activation, inflammatory signaling, endothelial function, and viral replication, supporting the concept that SARS‐CoV‐2 interferes with host epigenetic control mechanisms as part of its pathogenic strategy [19]. The reproducibility of these findings across cohorts suggests that miRNA dysregulation is not merely a bystander phenomenon but a central component of COVID‐19 pathophysiology.

A consistent observation across studies is the association between disease severity and distinct miRNA expression patterns. Mild or asymptomatic infection is generally characterized by increased levels of anti‐inflammatory and regulatory miRNAs, including miR‐21‐5p and miR‐29a‐3p, which may contribute to balanced immune responses and efficient viral clearance [20, 21]. In contrast, severe COVID‐19 is frequently associated with a global reduction in circulating miRNAs and marked dysregulation of key immunoregulatory molecules, particularly increased miR‐155‐5p and reduced miR‐146a‐5p [22, 23]. These miRNAs are known modulators of NF‐κB signaling, cytokine production, and endothelial activation, providing a mechanistic link between miRNA imbalance, hyperinflammation, vascular injury, and progression to ARDS. Together, these findings support the use of miRNA profiles as indicators of disease severity and prognostic biomarkers.

Interpretation of miRNA data, however, is highly dependent on sample source and timing. Early changes in miRNA expression likely reflect antiviral and innate immune responses, whereas later alterations may indicate immune exhaustion, tissue damage, or repair processes [21]. This temporal dimension partially explains inconsistencies reported across studies and highlights the importance of clinical context in miRNA‐based analyses.

Beyond free‐circulating miRNAs, extracellular vesicles (EVs) have emerged as critical mediators of miRNA‐driven intercellular communication during acute SARS‐CoV‐2 infection. EV‐associated miRNAs isolated from serum, plasma, or platelets show strong associations with inflammatory and thrombotic pathways. Elevated EV levels of miR‐20b‐5p, for example, were shown to induce neutrophil extracellular trap (NET) formation, directly linking miRNA cargo to immunothrombosis [24]. In severely ill patients, plasma EVs displayed differential expression of 50 miRNAs, with hsa‐miR‐1469 and hsa‐miR‐6124 demonstrating high prognostic accuracy for mortality prediction (AUC = 0.938) [25]. Platelet‐derived EVs enriched in miR‐21 and let‐7b were shown to activate TLR7/8 signaling in neutrophils, promoting ROS generation, cytokine release (IL‐1β, IL‐8, and TNF‐α), and NET formation [26]. Additionally, EVs from patients with pneumonia or ARDS induced expression of IL‐6, TLR4, and NLRP3 in recipient cells and carried tissue factor, further reinforcing the link between EV‐miRNAs, inflammation, and disease severity [27]. Collectively, these findings position EV‐associated miRNAs as both mechanistic drivers and promising prognostic biomarkers in acute COVID‐19.

Host miRNAs also regulate SARS‐CoV‐2 susceptibility by modulating the expression of viral entry receptors. Several studies have demonstrated post‐transcriptional control of ACE2 and TMPRSS2 by endogenous miRNAs [28]. miR‐200c‐3p negatively regulates ACE2 expression by binding to its 3^′^‐UTR, reducing receptor availability in pulmonary, renal, and cardiac tissues [28, 29]. While this mechanism may limit viral entry, it has also been associated with renin–angiotensin system imbalance and increased lung injury [30, 31]. Similarly, miR‐98‐5p suppresses TMPRSS2 expression in endothelial cells, suggesting that miRNA‐mediated receptor regulation contributes to interindividual variability in infection susceptibility and clinical outcomes [32].

The impact of miRNA dysregulation is further amplified in patients with pre‐existing comorbidities. Conditions such as diabetes, obesity, hypertension, and cardiovascular disease are associated with baseline alterations in miRNA expression that predispose individuals to exaggerated inflammatory responses during SARS‐CoV‐2 infection. Reduced miR‐146a levels, commonly observed in these conditions, remove a critical negative regulator of NF‐κB and toll‐like receptor signaling, favoring cytokine storm development and fibrosis [33]. In atherosclerotic cardiovascular disease, concomitant reduction of miR‐146a and elevation of miR‐27a link inflammation with lipid and glucose metabolism. In diabetic patients progressing to severe COVID‐19, increased miR‐34a‐5p—associated with cardiomyocyte apoptosis under hyperglycemic stress—has emerged as a potential prognostic biomarker [34]. These observations suggest that miRNAs not only reflect comorbidity status but also actively mediate mechanisms that exacerbate COVID‐19 severity.

In addition to host‐derived miRNAs, SARS‐CoV‐2 encodes viral miRNAs (v‐miRNAs) that further manipulate host cellular responses [35]. CoV2‐miR‐O7a, derived from the ORF7a gene, is processed independently of Drosha and incorporated into the RISC complex via AGO2 [36]. This v‐miRNA targets host genes, such as BATF2 and HSPG2, impairing innate immune activation and promoting immune evasion [35, 36]. Another v‐miRNA, CvmiR‐2, derived from delta and omicron variants, has been detected in patient samples and shown to target genes involved in immune regulation, fatigue resistance, and neurological function, with expression levels correlating with disease severity [37].

Beyond direct gene silencing, the viral genome itself may function as a miRNA sponge, sequestering host miRNAs including miR‐146a/b, miR‐21‐3p, miR‐30c‐5p, and miR‐374a‐3p [38]. This sequestration compromises host antiviral and anti‐inflammatory regulation, contributing to cytokine amplification and enhanced viral replication [38, 39]. Together, these mechanisms highlight a dual strategy by which SARS‐CoV‐2v‐miRNAs promote immune evasion and disease progression. Table 1 summarizes the dysregulated miRNAs in Acute COVID‐19.

**Table 1:** Dysregulated microRNAs in acute COVID‐19.

miRNA	Main function	Expression in COVID‐19	Clinical association	Source	Reference
miR‐21‐5p	Anti‐inflammatory	Upregulated in mild cases	Regulated immune response	Serum/plasma	[20, 21]
miR‐29a‐3p	Anti‐inflammatory, epigenetic regulator	Upregulated in mild cases	Balanced immune response	Serum	[20, 21]
miR‐155‐5p	Pro‐inflammatory	Upregulated in severe cases	Inflammation amplification	Plasma	[22, 23]
miR‐146a‐5p	Immunosuppressive, NF‐κB pathway regulator	Downregulated in severe/comorbid	Cytokine storm, fibrosis	EVs, tissues	[33]
miR‐20b‐5p	NET formation, thrombosis	Upregulated in EVs	Immunothrombosis	Serum EVs	[24]
miR‐1469	Prognostic marker	Upregulated in severe cases	AUC 0.938 for mortality prediction	Plasma EVs	[25]
miR‐200c‐3p	Negative regulator of ACE2	Upregulated in severe cases	Reduced ACE2, pulmonary injury	Lung cells	[28, 29]
miR‐98‐5p	Regulates TMPRSS2	Upregulated	Decreased susceptibility to infection	Endothelial cells	[32]
miR‐34a‐5p	Apoptosis in cardiomyocytes	Upregulated in severe diabetes	Prognostic biomarker	Cardiac tissue	[34]
CoV2‐miR‐O7a	Viral miRNA: silences host immune genes	Detected in severe cases	Suppressed antiviral response	ORF7a‐derived	[35, 36]
CvmiR‐2	Viral miRNA: variant‐specific (delta/omicron)	Elevated in severe cases	Immune evasion, neurological/fatigue‐related genes	Patient samples	[37]

Importantly, accumulating evidence indicates that miRNA dysregulation may persist beyond the acute phase, contributing to the development of LC. Distinct circulating miRNA signatures have been identified in patients with persistent symptoms, differentiating them from fully recovered individuals. Reduced plasma levels of miR‐200c‐3p, miR‐766‐3p, and miR‐142‐3p in patients with chronic rheumatologic symptoms suggest ongoing immune activation months after infection [18].

Longitudinal studies further support a role for miRNAs in post‐acute pulmonary dysfunction. In survivors of severe COVID‐19, miR‐9‐5p and miR‐486‐5p were independently associated with persistent reductions in pulmonary diffusion capacity (DLCO) up to 1 year after discharge [40]. These miRNAs, together with miR‐222‐3p, formed predictive signatures for prolonged respiratory impairment in machine learning models applied to ARDS survivors [41]. Complementary exosomal analyses revealed decreased miR‐17‐5p, miR‐146a‐5p, and miR‐223‐3p in patients with radiological lung sequelae, consistent with impaired resolution of inflammation and persistent NLRP3 inflammasome activation [42].

Systematic reviews further demonstrate overlap between miRNA profiles in LC and idiopathic pulmonary fibrosis, including increased pro‐fibrotic miRNAs (miR‐21‐5p, miR‐145‐5p, and miR‐199a‐5p) and reduced antifibrotic miRNAs (miR‐17‐5p, miR‐92a‐3p, and miR‐486‐5p), suggesting shared mechanisms of chronic tissue remodeling [43]. Neurological manifestations of LC have likewise been linked to dysregulation of multiple miRNAs involved in IL‐6/STAT3 signaling and barrier permeability, potentially underlying fatigue, headache, and neuropathic pain [44]. Reduced exosomal miR‐145 and miR‐885 levels associated with endothelial injury and thromboembolic risk further implicate miRNAs in the chronic vascular complications observed in post‐acute patients [45]. Table 2 summarizes the dysregulated miRNAs in LC.

**Table 2:** Dysregulated microRNAs in long COVID.

miRNA	Main function	Expression in long COVID	Clinical association	Source	Reference
miR‐200c‐3p	Inflammation, ACE2 regulation	Downregulated	Persistent inflammation	Plasma	[18]
miR‐766‐3p	Immune‐related pathways	Downregulated	Persistent inflammation	Plasma	[18]
miR‐142‐3p	Immune regulation	Downregulated	Persistent inflammation	Plasma	[18]
miR‐9‐5p	Inflammation, angiogenesis, senescence	Upregulated	Pulmonary dysfunction, reduced DLCO	Plasma	[40]
miR‐486‐5p	Angiogenesis, senescence, inflammation	Upregulated	Pulmonary fibrosis, reduced DLCO	Plasma	[40]
miR‐222‐3p	Respiratory impairment biomarker	Upregulated	Respiratory symptom persistence	ML model	[41]
miR‐17‐5p	Anti‐fibrotic, inflammation resolution	Downregulated	Failure to resolve inflammation	Exosomal	[42]
miR‐146a‐5p	Inflammation control, NLRP3 regulation	Downregulated	NLRP3 activation, inflammation	Exosomal	[42]
miR‐223‐3p	Inflammation control	Downregulated	Prolonged inflammatory state	Exosomal	[42]
miR‐21‐5p	Pro‐fibrotic, inflammation	Upregulated	Pulmonary fibrosis	Serum	[43]
miR‐145‐5p	Pro‐fibrotic	Upregulated	Fibrosis and tissue remodeling	Serum	[43]
miR‐199a‐5p	Pro‐fibrotic	Upregulated	Fibrosis	Serum	[43]
miR‐92a‐3p	Anti‐fibrotic	Downregulated	Loss of repair capacity	Serum	[43]
miR‐29a/b/c‐3p	Fibrosis, inflammation	Upregulated	Chronic inflammation, fibrosis	Serum	[43]
miR‐126‐3p	Vascular homeostasis	Upregulated	Vascular dysfunction	Serum	[43]
miR‐150‐5p	Immune regulation	Upregulated	Immune dysregulation	Serum	[43]
miR‐155‐5p	Pro‐inflammatory	Upregulated	Neuroinflammation	Serum	[43]
miR‐200a/c‐3p	Immune regulation, fibrosis	Upregulated	Tissue remodeling, fibrosis	Serum	[43]
miR‐320a/b/c/d/e‐3p	Neurological inflammation	Upregulated	Neurological symptoms (fatigue, pain)	Serum	[43]
miR‐451a	Blood–nerve barrier regulation	Upregulated	Neuroinflammation, IL‐6 signaling	Serum	[43]
miR‐145	Endothelial integrity, fibrosis	Downregulated	Endothelial damage, fibrotic remodeling	Exosomes	[45]
miR‐885	Thrombosis, endothelial apoptosis	Downregulated	Thrombosis, impaired angiogenesis	Exosomes	[45]

Despite the growing number of studies examining dysregulated miRNAs in acute COVID‐19 and LC, the literature still shows important limitations. Most evidence derives from the acute phase of SARS‐CoV‐2 infection, while longitudinal analyses of miRNA dynamics are scarce, making it difficult to distinguish between transient inflammatory responses from persistent alterations linked to LC. In addition, many studies involve small cohorts, heterogeneous patient populations, and non‐standardized methodologies. Differences in sample type, timing of collection, and clinical variables contribute to inconsistent results, as illustrated by conflicting reports on miR‐146a‐5p. Functional validation is also limited, with most findings based on bioinformatic predictions and correlations rather than on mechanistic experiments.

Research on LC is particularly limited. Only a few studies have profiled miRNAs in post‐acute patients, and even fewer have included longitudinal follow‐up or robust control for confounding factors. Although some miRNA signatures show potential as biomarkers, their clinical utility remains unconfirmed. The underrepresentation of diverse populations further restricts generalizability.

## 4. Conclusion

**Figure 3:**
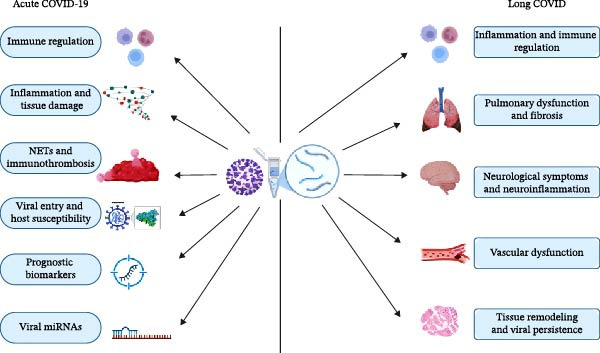
Proposed roles of microRNAs in the pathophysiology of acute COVID‐19 and long COVID. This image depicts the regulatory roles of microRNAs (miRNAs) during the acute and post‐acute phases of COVID‐19. It highlights how specific miRNAs modulate host immune responses, inflammation, and tissue repair processes. In acute infection, miRNAs may influence viral replication and cytokine production, while in LC, persistent miRNA dysregulation is associated with chronic inflammation, immune dysfunction, and unresolved symptoms. The illustration underscores the potential of miRNAs as biomarkers and therapeutic targets across different stages of the disease.

This review highlights miRNAs as key post‐transcriptional regulators shaping the host response to SARS‐CoV‐2 infection, from acute disease to the persistent manifestations of LC. Accumulating evidence indicates that dysregulated host and v‐miRNAs—particularly those associated with EVs—modulate immune activation, inflammation, endothelial dysfunction, and tissue remodeling, as can be seen in Figure 3, supporting their relevance beyond biomarker discovery. However, despite emerging miRNA signatures linked to post‐acute pulmonary, neurological, and vascular sequelae, research in LC remains limited and methodologically heterogeneous, with a strong bias toward acute‐phase analyses. Consequently, longitudinal and mechanistically driven studies are urgently needed to distinguish residual molecular alterations from active drivers of chronic disease. Integrating standardized miRNA profiling with clinical phenotyping may accelerate the identification of robust biomarkers and therapeutic targets, strengthening the translational impact of miRNA research in addressing the long‐term burden of COVID‐19.

## Funding

This study was financed in part by the Coordenação de Aperfeiçoamento de Pessoal de Nível Superior, Brasil (CAPES; Finance Code 001, master fellowship awarded to Larissa I. Silva).

## Conflicts of Interest

The authors declare no conflicts of interest.

## Data Availability

Data sharing is not applicable to this article, as no datasets were generated or analyzed during the current study.

## References

[1] He X. , Lau E. H. Y. , and Wu P. , et al.Temporal Dynamics in Viral Shedding and Transmissibility of COVID-19, Nature Medicine. (2020) 26, no. 5, 672–675, 10.1038/s41591-020-0869-5. 32296168

[2] Lopez-Leon S. , Wegman-Ostrosky T. , and Perelman C. , et al.More than 50 Long-Term Effects of COVID-19: A Systematic Review and Meta-Analysis, Scientific Reports. (2021) 11, no. 1, 10.1038/s41598-021-95565-8, 16144. 34373540 PMC8352980

[3] Mahdi M. , Kiarie I. W. , and Mótyán J. A. , et al.Receptor Binding for the Entry Mechanisms of SARS-CoV-2: Insights From the Original Strain and Emerging Variants, Viruses. (2025) 17, no. 5, 10.3390/v17050691, 691. 40431702 PMC12115909

[4] Harvey W. T. , Carabelli A. M. , and Jackson B. , et al.SARS-CoV-2 Variants, Spike Mutations and Immune Escape, Nature Reviews Microbiology. (2021) 19, no. 7, 409–424, 10.1038/s41579-021-00573-0. 34075212 PMC8167834

[5] Sliwa K. , Singh K. , and Nikhare K. , et al.Long COVID Syndrome, Mortality and Morbidity in Patients Hospitalized With COVID-19 From 16 Countries: The World Heart Federation Global COVID-19 Study, Global Heart. (2025) 20, no. 1, 10.5334/gh.1452, 66. 40757253 PMC12315686

[6] Li G. , Fan Y. , and Lai Y. , et al.Coronavirus Infections and Immune Responses, Journal of Medical Virology. (2020) 92, no. 4, 424–432, 10.1002/jmv.25685. 31981224 PMC7166547

[7] Jackson C. B. , Farzan M. , Chen B. , and Choe H. , Mechanisms of SARS-CoV-2 Entry Into Cells, Nature Reviews Molecular Cell Biology. (2022) 23, no. 1, 3–20, 10.1038/s41580-021-00418-x. 34611326 PMC8491763

[8] Lebedin M. , Ratswohl C. , and Garg A. , et al.Soluble ACE2 Correlates With Severe COVID-19 and Can Impair Antibody Responses, iScience. (2024) 27, no. 3, 10.1016/j.isci.2024.109330, 109330. 38496296 PMC10940809

[9] Venter C. , Bezuidenhout J. A. , and Laubscher G. J. , et al.Erythrocyte, Platelet, Serum Ferritin, and P-Selectin Pathophysiology Implicated in Severe Hypercoagulation and Vascular Complications in COVID-19, International Journal of Molecular Sciences. (2020) 21, no. 21, 10.3390/ijms21218234, 8234. 33153161 PMC7662625

[10] Del Valle D. M. , Kim-Schulze S. , and Huang H. H. , et al.An Inflammatory Cytokine Signature Predicts COVID-19 Severity and Survival, Nature Medicine. (2020) 26, no. 10, 1636–1643, 10.1038/s41591-020-1051-9. PMC7869028 32839624

[11] Bastard P. , Rosen L. B. , and Zhang Q. , et al.Autoantibodies Against Type I IFNs in Patients With Life-Threatening COVID-19, Science. (2020) 370, no. 6515, 10.1126/science.abd4585. PMC7857397 32972996

[12] Severe Covid-19 GWAS Group , Genomewide Association Study of Severe Covid-19 With Respiratory Failure, New England Journal of Medicine. (2020) 383, no. 16, 1522–1534, 10.1056/NEJMoa2020283. 32558485 PMC7315890

[13] Lukiw W. J. , MicroRNA Heterogeneity, Innate-Immune Defense and the Efficacy of SARS-CoV-2 Infection—A Commentary, Non-Coding RNA. (2021) 7, no. 2, 10.3390/ncrna7020037, 37. 34207055 PMC8293307

[14] O’Brien J. , Hayder H. , Zayed Y. , and Peng C. , Overview of MicroRNA Biogenesis, Mechanisms of Actions, and Circulation, Frontiers in Endocrinology. (2018) 9, 10.3389/fendo.2018.00402, 2-s2.0-85054929695. PMC6085463 30123182

[15] Jankovic M. , Nikolic D. , Novakovic I. , Petrovic B. , Lackovic M. , and Santric-Milicevic M. , miRNAs as a Potential Biomarker in the COVID-19 Infection and Complications Course, Severity, and Outcome, Diagnostics. (2023) 13, no. 6, 10.3390/diagnostics13061091, 1091. 36980399 PMC10047241

[16] Condrat C. E. , Thompson D. C. , and Barbu M. G. , et al.miRNAs as Biomarkers in Disease: Latest Findings Regarding Their Role in Diagnosis and Prognosis, Cells. (2020) 9, no. 2, 10.3390/cells9020276, 276. 31979244 PMC7072450

[17] Liang Y. , Fang D. , and Gao X. , et al.Circulating MicroRNAs as Emerging Regulators of COVID-19, Theranostics. (2023) 13, no. 1, 125–147, 10.7150/thno.78164. 36593971 PMC9800721

[18] Timofeeva A. M. , Nikitin A. O. , and Nevinsky G. A. , Circulating miRNAs in the Plasma of Post-COVID-19 Patients With Typical Recovery and Those With Long-COVID Symptoms: Regulation of Immune Response-Associated Pathways, Non-Coding RNA. (2024) 10, no. 5, 10.3390/ncrna10050048, 48. 39311385 PMC11417918

[19] Yang S. , Wu S. , and Yu Z. , et al.Transcriptomic Analysis Reveals Novel Mechanisms of SARS-CoV-2 Infection in Human Lung Cells, Immunity, Inflammation and Disease. (2020) 8, no. 4, 753–762, 10.1002/iid3.366. 33124193 PMC7654422

[20] de Gonzalo-Calvo D. , Benítez I. D. , and Pinilla L. , et al.Circulating microRNA Profiles Predict the Severity of COVID-19 in Hospitalized Patients, Translational Research. (2021) 236, 147–159, 10.1016/j.trsl.2021.05.004. 34048985 PMC8149473

[21] Farr R. J. , Rootes C. L. , and Rowntree L. C. , et al.Altered microRNA Expression in COVID-19 Patients Enables Identification of SARS-CoV-2 Infection, PLoS Pathogens. (2021) 17, no. 7, 10.1371/journal.ppat.1009759, e1009759. 34320031 PMC8318295

[22] Soltani-Zangbar M. S. , Hajivalili M. , and Daneshdoust D. , et al.SARS-CoV2 Infection Induce miR-155 Expression and Skewed Th17/Treg Balance by Changing SOCS1 Level: A Clinical Study, Cytokine. (2023) 169, 10.1016/j.cyto.2023.156248, 156248. 37307689 PMC10247889

[23] Sabbatinelli J. , Giuliani A. , and Matacchione G. , et al.Decreased Serum Levels of the Inflammaging Marker miR-146a Are Associated With Clinical Non-Response to Tocilizumab in COVID-19 Patients, Mechanisms of Ageing and Development. (2021) 193, 10.1016/j.mad.2020.111413, 111413. 33307107 PMC7722494

[24] Liao Y. , Liu Y. , and Li D. , et al.COVID-19 Patient Serum-Derived Extracellular Vesicles Deliver miR-20b-5p Induces Neutrophil Extracellular Traps, Cell Communication and Signaling. (2025) 23, no. 1, 10.1186/s12964-025-02095-1, 93. 39962581 PMC11834185

[25] Sánchez-de Prada L. , García-Concejo A. , and Tamayo-Velasco Á. , et al.miRNome Profiling of Extracellular Vesicles in Patients With Severe COVID-19 and Identification of Predictors of Mortality, The Journal of Infectious Diseases. (2024) 12, 10.1093/infdis/jiae310. 38865487

[26] Liao T.-L. , Liu H.-J. , Chen D.-Y. , Tang K.-T. , Chen Y.-M. , and Liu P.-Y. , SARS-CoV-2 Primed Platelets–Derived microRNAs Enhance NETs Formation by Extracellular Vesicle Transmission and TLR7/8 Activation, Cell Communication and Signaling. (2023) 21, no. 1, 10.1186/s12964-023-01345-4, 304. 37904132 PMC10614402

[27] Nair S. , Nova-Lamperti E. , and Labarca G. , et al.Genomic Communication via Circulating Extracellular Vesicles and Long-Term Health Consequences of COVID-19, Journal of Translational Medicine. (2023) 21, no. 1, 10.1186/s12967-023-04552-2, 709. 37817137 PMC10563316

[28] Wang C.-W. , Chuang H.-C. , and Tan T.-H. , ACE2 in Chronic Disease and COVID-19: Gene Regulation and Post-Translational Modification, Journal of Biomedical Science. (2023) 30, no. 1, 10.1186/s12929-023-00965-9, 71. 37608279 PMC10464117

[29] Bellae Papannarao J. , Schwenke D. O. , Manning P. , and Katare R. , Upregulated miR-200c Is Associated With Downregulation of the Functional Receptor for Severe Acute Respiratory Syndrome Coronavirus 2 ACE2 in Individuals With Obesity, International Journal of Obesity. (2022) 46, no. 1, 238–241, 10.1038/s41366-021-00984-2. 34625660 PMC8499608

[30] Zou X. , Chen K. , Zou J. , Han P. , Hao J. , and Han Z. , Single-Cell RNA-Seq Data Analysis on the Receptor ACE2 Expression Reveals the Potential Risk of Different Human Organs Vulnerable to 2019-nCoV Infection, Frontiers of Medicine. (2020) 14, no. 2, 185–192, 10.1007/s11684-020-0754-0. 32170560 PMC7088738

[31] Hoffmann M. , Kleine-Weber H. , and Schroeder S. , et al.SARS-CoV-2 Cell Entry Depends on ACE2 and TMPRSS2 and Is Blocked by a Clinically Proven Protease Inhibitor, Cell. (2020) 181, no. 2, 271–280.e8, 10.1016/j.cell.2020.02.052. 32142651 PMC7102627

[32] Matarese A. , Gambardella J. , Sardu C. , and Santulli G. , miR-98 Regulates TMPRSS2 Expression in Human Endothelial Cells: Key Implications for COVID-19, Biomedicines. (2020) 8, no. 11, 10.3390/biomedicines8110462, 462. 33143053 PMC7693865

[33] Roganović J. , Downregulation of microRNA-146a in Diabetes, Obesity and Hypertension May Contribute to Severe COVID-19, Medical Hypotheses. (2021) 146, 10.1016/j.mehy.2020.110448, 110448. 33338955 PMC7836676

[34] Khatami A. , Taghizadieh M. , and Sadri Nahand J. , et al.Evaluation of MicroRNA Expression Pattern (miR-28, miR-181a, miR-34a, and miR-31) in Patients With COVID-19 Admitted to ICU and Diabetic COVID-19 Patients, Intervirology. (2023) 63–76, 10.1159/000529985. 36882006 PMC10308556

[35] Pawlica P. , Yario T. A. , and White S. , et al.SARS-CoV-2 Expresses a microRNA-Like Small RNA Able to Selectively Repress Host Genes, Proceedings of the National Academy of Sciences. (2021) 118, no. 52, 10.1073/pnas.2116668118. PMC8719879 34903581

[36] Singh M. , Chazal M. , and Quarato P. , et al.A Virus-Derived microRNA Targets Immune Response Genes During SARS-CoV-2 Infection, EMBO Reports. (2022) 23, no. 2, 10.15252/embr.202154341. PMC8811647 34914162

[37] Zhao Q. , Lü J. , and Zhao B. , et al.Identification of a SARS-CoV-2 Virus-Derived vmiRNA in COVID-19 Patients Holding Potential as a Diagnostic Biomarker, Frontiers in Cellular and Infection Microbiology. (2023) 13, 10.3389/fcimb.2023.1190870. PMC10272551 37333844

[38] Li C. , Wang R. , and Wu A. , et al.SARS-COV-2 as Potential microRNA Sponge in COVID-19 Patients, BMC Medical Genomics. (2022) 15, no. S2, 10.1186/s12920-022-01243-7, 94. 35461273 PMC9034446

[39] Yang C.-Y. , Chen Y.-H. , Liu P.-J. , Hu W.-C. , Lu K.-C. , and Tsai K.-W. , The Emerging Role of miRNAs in the Pathogenesis of COVID-19: Protective Effects of Nutraceutical Polyphenolic Compounds Against SARS-CoV-2 Infection, International Journal of Medical Sciences. (2022) 19, no. 8, 1340–1356, 10.7150/ijms.76168. 35928726 PMC9346380

[40] García-Hidalgo M. C. , Benítez I. D. , and Perez-Pons M. , et al.MicroRNA-Guided Drug Discovery for Mitigating Persistent Pulmonary Complications in Critical COVID-19 Survivors: A Longitudinal Pilot Study, British Journal of Pharmacology. (2025) 182, no. 2, 380–395, 10.1111/bph.16330. 38359818

[41] García-Hidalgo M. C. , González J. , and Benítez I. D. , et al.Identification of Circulating microRNA Profiles Associated With Pulmonary Function and Radiologic Features in Survivors of SARS-CoV-2-Induced ARDS, Emerging Microbes & Infections. (2022) 11, no. 1, 1537–1549, 10.1080/22221751.2022.2081615. 35603455 PMC9176679

[42] Curcio R. , Poli G. , and Fabi C. , et al.Exosomal miR-17-5p, miR-146a-3p, and miR-223-3p Correlate With Radiologic Sequelae in Survivors of COVID-19-Related Acute Respiratory Distress Syndrome, International Journal of Molecular Sciences. (2023) 24, no. 17, 10.3390/ijms241713037, 13037. 37685844 PMC10488112

[43] Guiot J. , Henket M. , and Remacle C. , et al.Systematic Review of Overlapping microRNA Patterns in COVID-19 and Idiopathic Pulmonary Fibrosis, Respiratory Research. (2023) 24, no. 1, 10.1186/s12931-023-02413-6, 112. 37061683 PMC10105547

[44] Reyes-Long S. , Cortés-Altamirano J. L. , and Bandala C. , et al.Role of the MicroRNAs in the Pathogenic Mechanism of Painful Symptoms in Long COVID: Systematic Review, International Journal of Molecular Sciences. (2023) 24, no. 4, 10.3390/ijms24043574, 3574. 36834984 PMC9963913

[45] Gambardella J. , Kansakar U. , and Sardu C. , et al.Exosomal miR-145 and miR-885 Regulate Thrombosis in COVID-19, The Journal of Pharmacology and Experimental Therapeutics. (2023) 384, no. 1, 109–115, 10.1124/jpet.122.001209. 35772782 PMC9827505

